# Unmasking symptomatic dermatographism in the time of COVID-19

**DOI:** 10.1136/postgradmedj-2020-138688

**Published:** 2020-09-10

**Authors:** Katelyn H Wong, Thomas Murray, Rachel Osborn, Gary K Soffer

**Affiliations:** Pediatrics, Yale School of Medicine, New Haven, Connecticut, USA; Section of Pediatric Infectious Diseases, Department of Pediatrics, Yale School of Medicine, New Haven, Connecticut, USA; Pediatrics, Yale School of Medicine, New Haven, Connecticut, USA; Section of Allergy and Immunology, Department of Pediatrics, Yale School of Medicine, New Haven, Connecticut, USA

**Figure 1:**
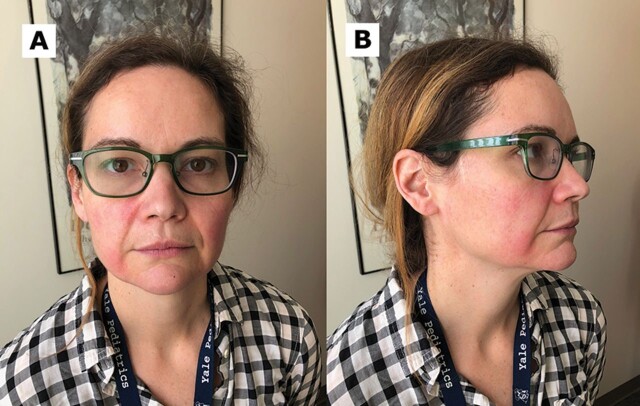
Facial urticaria limited to area occluded by N95 respirator (a, b).

**Figure 2:**
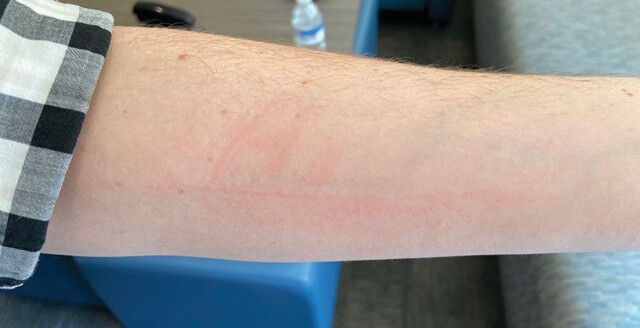
Firm stroking of the skin produced new linear wheals.

A 39-year-old woman presented with localized urticaria following N95 respirator use in a COVID-19 unit. After approximately 15 minutes of wear, she developed pruritus on her face, without associated angioedema or dyspnea. She had no history of cutaneous reactions to N95 respirators. Physical examination revealed several wheals with surrounding erythema limited to the area occluded by the N95 respirator (figure 1). On evaluation, firm stroking of the volar aspect of her forearm produced linear wheals (figure 2); she was subsequently diagnosed with dermatographism. Symptoms resolved within 1 hour of antihistamine use.

Dermatographism is characterised by the development of a wheal and flare reaction after pressure is applied to the skin by stroking or scratching, typically within minutes and persisting up to an hour.^1^ Symptoms typically self-resolve within an hour. Antihistamines can be used in symptomatic dermatographism. The pathogenesis is likely related to vasoactive mediators released by mast cells as elevated serum levels of histamine have been associated.^2^ Differential diagnoses including pressure urticaria and contact dermatitis can be ruled out based on inciting factor, timing of onset and resolution with antihistamines.

N95 respirators are recommended for healthcare workers (HCWs) who provide care for patients with suspected or confirmed COVID-19 infection.^3^ The recent pandemic has led to increased and extended respirator use. While evidence supports these respirators as offering substantial protection against COVID-19, this case highlights that occupational health departments should be alert for adverse cutaneous reactions that may affect HCWs.^4^
